# Unlocking the Potential of Saliva-Based Test to Detect HPV-16-Driven Oropharyngeal Cancer

**DOI:** 10.3390/cancers11040473

**Published:** 2019-04-03

**Authors:** Kai Dun Tang, Kurt Baeten, Liz Kenny, Ian H. Frazer, Gert Scheper, Chamindie Punyadeera

**Affiliations:** 1 The School of Biomedical Sciences, Institute of Health and Biomedical Innovation, Queensland University of Technology and the Translational Research Institute, Queensland 4059, Australia; kai.tang@qut.edu.au; 2 Janssen Diagnostics, Janssen Pharmaceutica NV, Beerse 2340, Belgium; KBAETEN1@its.jnj.com; 3 School of Medicine, University of Queensland, Queensland 4029, Australia; lizkenny@bigpond.net.au; 4 Central Integrated Regional Cancer Services, Royal Brisbane and Women’s Hospital, Queensland Health, Queensland 4029, Australia; 5 Faculty of Medicine, The University of Queensland, Translational Research Institute, Queensland 4102, Australia; i.frazer@uq.edu.au; 6 Janssen Vaccines & Prevention BV, Leiden 2333, The Netherlands; GSCHEPER@its.jnj.com

**Keywords:** human papillomavirus, oropharyngeal cancer, saliva, HPV-16 viral load, HPV-16 integration

## Abstract

The incidence of human papillomavirus (HPV)-positive oropharyngeal cancer (OPC) is rising in high-income countries, including Australia. Increasing evidence suggests that accurate HPV testing is pivotal for clinical decision making and treatment planning in these patients. Recently, the eighth edition of the American Joint Committee on Cancer/Union for International Cancer Control (AJCC/UICC) tumor–node–metastasis (TNM) staging system for OPC (based on the p16INK4a (p16) status) was proposed and has been implemented. However, the applicability of this new staging system is still far from clear. In our study, *n* = 127 OPC patients from Queensland, Australia were recruited, and the tumor p16 expression in these patients was examined using immunohistochemical (IHC) analysis. HPV-16 genotyping, viral load, and physical status (episomal versus integrated) in the saliva samples of OPC patients were determined using the qPCR method. A good inter-rater agreement (*k* = 0.612) was found between tumor p16 expression and oral HPV-16 infection in OPC. Importantly, according to the eighth edition staging system, HPV-16 DNA viral load (>10 copies/50 ng) was significantly associated with the advanced stages of OPC. In concordance with previous studies, a mixed HPV-16 form (partially or fully integrated) was predominately found in OPC patients. Taken together, our data support HPV-16 detection in saliva as a screening biomarker to identify people within the community who are at risk of developing OPC.

## 1. Introduction

Oropharyngeal cancer (OPC) usually arises from the tonsillar area, the base of tongue, and the oropharynx, and is one of the most common subtypes of head and neck cancers (HNC), accounting for 97,000 deaths annually worldwide [1,2]. There has been a significant increase in the incidence of high-risk human papillomavirus (HPV)-associated OPC in high-income countries (predominantly HPV-16), when compared to HPV-negative HNC [3,4]In Australia, the prevalence rate of HPV-positive cases among all OPC cases increased from 20.2% to 63.5% over the past two decades [3]. Strikingly, it is projected to surpass cervical cancer incidence by 2020 in the United States (USA) [4]. Factors associated with the rising of OPC incidence include a large number of lifetime sexual partners, sexual behavior (e.g., oral sex), poor oral health/hygiene, and being a partner of patients with HPV-related cancers [5,6,7].

The clinical diagnosis of OPC is challenging when compared to other cancer types such as prostate and breast, as the tumors are usually tiny in the early stages of disease, and are located in the regions of the mouth that are not easily visible and accessible [8,9]. As a consequence, most of them are either misdiagnosed or only diagnosed at an advanced stage, which leads to complicated treatments. Treatment in an advanced-stage disease is often associated with a reduction in cure rate as well as significant sequelae with a high impact on the quality of life, which can include difficulty in swallowing (dysphagia) and speech problems. Importantly, HPV-positive OPC is linked with a more favorable prognosis, but with high recurrences within two years of diagnosis when compared to HPV-negative disease [10,11]; therefore, an accurate and early diagnosis is essential to reduce the disease burden.

In January 2018, the eighth edition of the American Joint Committee on Cancer/Union for International Cancer Control (AJCC/UICC) tumor–node–metastasis (TNM) staging system for OPC was released and adopted by the clinicians. In this edition, OPC patients are stratified based on the tumor p16INK4a (p16) status, which is a common surrogate marker for HPV [12]. However, p16 as a standalone marker for the detection of HPV in OPC patients has been shown to have limitations, resulting in false positivity in 10–20% of cases [13,14]. Previous studies have demonstrated the importance of additional HPV DNA testing in diagnosis and prognosis, as well as tailoring treatment to the individual patients [15,16].

There is solid evidence to support the use of saliva as a nexus diagnostic testing fluid for the detection of HPV DNA in OPC patients [17,18]. Furthermore, the detection of HPV DNA in saliva collected by different methods (drool or oral rinse) yields comparable results and shows good sensitivity for the detection of HPV, again supporting the feasibility of using saliva as a diagnostic medium for OPC [19]. In this study, we aimed to investigate the oral HPV-16 prevalence, viral load, and physical status in a cohort of Australian patients with OPC classified based on the seventh and eighth edition of the AJCC/UICC TNM staging system.

## 2. Results

### 2.1. Patient Characteristics

The demographics of OPC patients (*n* = 121) who could provide a sufficient amount of DNA are summarized in Table 1. Most of the patients were male (91%) and over 55 years of age (74%). The majority of them were considered as ever-smokers (74%). OPC tumors were predominantly found in the tonsillar region (50%). p16 immunohistochemistry (IHC) analysis was scored positive in 89 out of 121 OPC patients (74%).

**Figure 1:**
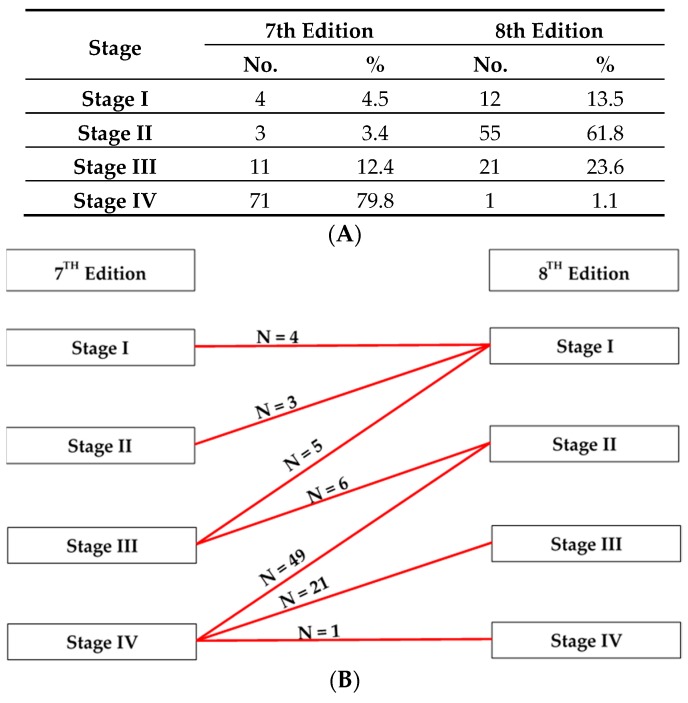
Distribution of disease stage in 89 p16INK4a (p16)-positive oropharyngeal cancer (OPC) patients. (**A**) Comparison between the seventh and eighth edition staging system for p16-positive OPC. (**B**) Most of the seventh-edition Stage IV tumors were downgraded to Stage II and III according to the eighth edition staging system.

A comparison of the seventh and eighth edition staging systems for p16-positive OPC patients is listed in Figure 1A,B. When the eighth edition staging system was applied, 100% and 45% of the seventh-edition Stage II and Stage III tumors were respectively downgraded to Stage I, while 55% and 69% of the of seventh-edition Stage III and Stage IV tumors were respectively downgraded to Stage II, and 30% of the seventh-edition Stage IV tumors were downgraded to Stage III.

### 2.2. Tumor p16 Expression and Oral HPV-16 Infection in OPC

Salivary HPV-16 DNA (E2 or/and E6/7) was detected in 71 out of 89 (80%) p16-positive OPC patients, as shown in Table 2. The inter-rater agreement between oral HPV-16 infection and tumor p16 expression was considered as good (*k* = 0.612, 95% CI: 0.468, 0.756). All of the samples were positive for beta-goblin

### 2.3. Viral Load of HPV-16 in OPC

**Figure 2:**
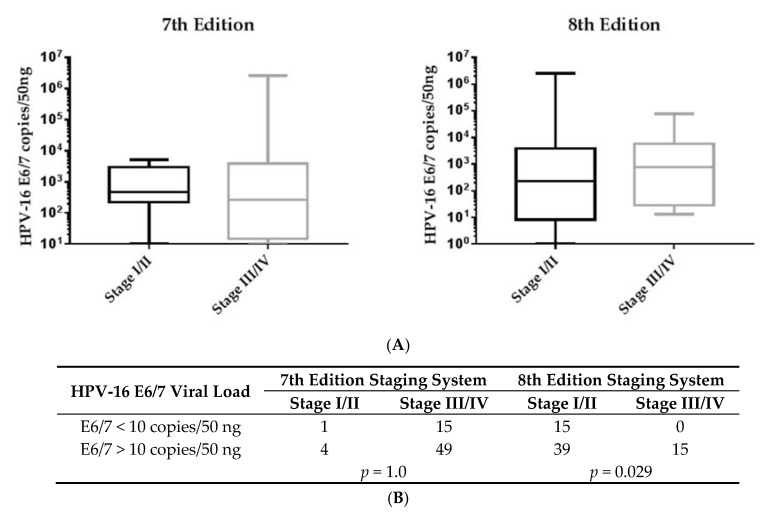
Salivary human papillomavirus (HPV)-16 E6/7 viral load in OPC patients. (**A**) The medium value of the HPV-16 E6/7 viral load showed a trend of being higher in advanced stages (III/IV) when compared to early stages (I/II) of OPC according to the eighth edition staging system. (**B**) Similarly, based on the eighth edition staging system, salivary HPV-16 viral load (>10 copies/50 ng) was significantly associated with advanced stages OPC.

When tumors were classified by the seventh edition staging system, early stages (I and II) of OPC showed a higher viral load with a median value of 475.4 copies of HPV-16 E6/7 DNA per 50 ng when compared to advanced stages (III and IV) (266.5 copies/50 ng). Conversely, when the eighth edition staging system was used, the HPV-16 viral load was elevated in advanced stages of OPC (774.1 copies/50 ng) when compared to early stages (232.0 copies/50 ng) (Figure 2A). More importantly, based on the eighth edition staging system, there was a significant positive correlation between the HPV-16 viral loads (>10 copies/50 ng) and disease stage of OPC, as shown in Figure 2B.

### 2.4. Physical Status of HPV-16 in OPC

The oral HPV-16 physical status (episomal, integrated, and mixed) in 71 OPC patients was examined by qPCR-based E2/E6/7 ratios, as shown in Table 3. Partially or fully HPV-16 integrated forms (when the E2/E6/7 ratio was equal to 0 or/and between 0–1) were commonly found in both early and advanced stages of OPC patients according to the seventh and eighth edition staging systems. Furthermore, there was no significant difference between the partially or fully HPV-16 integrated form and the disease stage of OPC patients.

## 3. Discussion

The incidence of HPV-positive OPC, particularly in younger men, is accelerating in high-income countries as well as in Queensland and Australia [20,21]. Recently, the eighth edition staging system for OPC was proposed and has been implemented. However, the applicability of this staging system has not been fully established yet. In this study, we showed a significant difference in the distribution of disease stage for p16-positive OPC between the seventh and eighth edition staging system in an Australian patient cohort. Consistent with previous research, we demonstrated a good inter-rater agreement between tumor p16 expression and oral HPV-16 infection. Interestingly, an elevated HPV-16 viral load (>10 copies/50 ng) was significantly associated with the advanced stages of OPC based on the eighth edition staging system. However, neither the eighth edition nor the seventh edition staging system showed a positive association between HPV-16 E2/E6/7 ratio and the risk of OPC.

Increasing evidence supports the notion that an accurate HPV test is crucial for clinical decision making and treatment planning for OPC patients [22,23,24]. HPV testing on tumor biopsy using p16 staining is considered as the standard of care for OPC patients worldwide. In comparison with the seventh edition, the eighth edition staging system based on p16 status seems to have a better predictive prognostic power for p16-positive OPC [15]. However, Nauta et al. reported that HPV DNA-negative but p16-positive OPC patients had a worse prognosis than patients with HPV DNA-positive OPC [15]. Another recent study also showed that the overall survival was significantly lower in patients with p16-positive/HPV-negative OPC when compared with the group in which both p16 and HPV DNA were positive [14], further reinforcing the importance of performing additional HPV DNA testing in OPC patients.

At present, there is no standard or routine HPV DNA testing for OPC; therefore, it is pivotal to develop a non-invasive and low-cost test. The results of several studies support the idea of using salivary HPV DNA as a biomarker to monitor disease progression and tumor recurrence in OPC patients [22,25,26]. Previous studies demonstrated that the presence of HPV DNA in tumor tissues and plasma was significantly correlated with HPV DNA positivity in saliva samples collected from OPC patients [19,22,25]. In concordance with previously published data [13,14,15], we demonstrate that approximately 20% of p16-positive patients were negative for salivary HPV-16 DNA, which raises the possibility of using saliva as a diagnostic medium for HPV testing in OPC patients.

The persistence of high-risk HPV infections (HPV-16 and 18) is strongly associated with the development of cervical neoplasia and progression toward cervical carcinoma [27,28]. In addition, the association between HPV infection and the development of OPC has been postulated several years ago, and was most convincingly demonstrated by Agalliu et al. [29]. However, in contrast to cervical carcinoma patients, there have been several studies in the literature reporting a large variation in HPV-16 viral loads among OPC patients [30,31]. Strikingly, our results revealed a statistical significance between salivary HPV-16 viral load (>10 copies/50 ng) and the disease stage of OPC based on the eighth edition staging system, suggesting the prognostic value of salivary HPV-16 DNA in OPC.

Persistent high-risk HPV infections can also trigger genomic instability and subsequently promote the integration of viral DNA into the host genome [32,33]. This may result in a disruption of the E2 open reading frame (ORF), which plays a major role in regulating the activity of the viral promoter. The loss of E2 expression contributes to the upregulation of E6 and E7 oncogenic protein, and hence promotes the cancer development and progression [34]. This is further supported by previous studies that indicated that partially or fully integrated HPV-16 is commonly detected in cervical carcinoma biopsies [35]. These results were in concordance with our findings in OPC patients. However, no relationship between the HPV-16 integration and disease stage of OPC based on either the seventh or eighth edition staging systems was observed. The limitations to this study are: a single-site recruitment (only restricted to Queensland), the modest sample size of OPC patients, and the limited availability of fresh tumor samples. Larger multicenter studies from various geographic regions and additional techniques for the examination of salivary HPV-16 physical status may overcome these shortcomings.

## 4. Materials and Methods

### 4.1. Study Design

**Figure 3:**
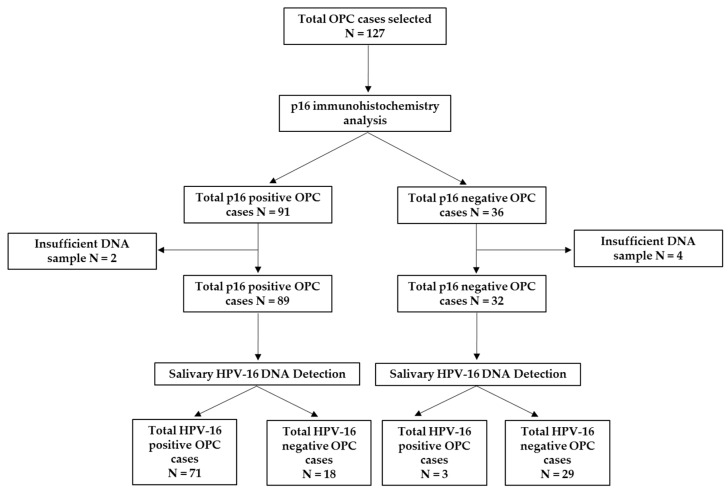
Flow chart of study recruitment.

This study was approved by the University of Queensland (UQ) Medical Ethical Institutional Board (HREC No: 2014000679 and 2014000862); Queensland University of Technology (QUT) (HREC No: 1400000617 and 1400000641); the Princess Alexandra Hospital (PAH) Ethics Review Board (HREC/12/QPAH/381), and the Royal Brisbane and Women′s Hospital (RBWH) (HREC/16/QRBW/447). A total of 127 patients who have been diagnosed with OPC from the PAH and RBWH were recruited to this study (Figure 3). All of the participants provided written informed consent prior to collecting samples. Clinical stages of OPC patients were classified according to the seventh and eighth edition of the AJCC/UICC TNM staging system. In addition, p16 IHC was used to evaluate the HPV status in OPC patients.

### 4.2. Saliva and Oral Rinses Collection and Processing

Saliva and oral rinse samples of participants were collected as previously described [36]. Briefly, participants were asked to rinse their mouths with drinking water to get rid of all the food debris before the sample collection. For saliva samples, participants were asked to tilt their heads down and gather the saliva in the mouth for 2 to 5 min before drooling into a sterile 50-mL falcon tube. For oral rinse samples, participants were asked to swish and gargle for 1 to 2 min with 2 × 10 mL 0.9% saline before expectorating into a 50-mL falcon tube. All of the samples were immediately placed on dry ice and transported to the QUT laboratory for downstream processing.

### 4.3. DNA Extraction

Total DNA was isolated from saliva/oral rinse samples using the QIAamp DNA Mini Kit (Qiagen, Germantown, MD, USA), following the manufacturer’s instructions. Briefly, cell pellets were resuspended with 200 μL of phosphate-buffered saline (PBS) before adding the 10 μL of proteinase K and 200 μL of lysis buffer to the mixture. After 10 min of incubation at 56 °C, 200 μL of 100% ethanol was added to the mixture, which was then transferred to QIAmp DNA mini spin columns as per manufacturer protocol.

### 4.4. qPCR Analysis

For the detection of HPV-16 positively in saliva samples, qPCR was carried out with the specific primers targeted against the HPV-16 E2 (Forward: AACGAAGTATCCTCTCCTGAAATTATTAG; Reverse: CCAAGGCGACGGCTTTG) and E6/7 (Forward: ACCGGTCGATGTATGTCTTGTTG; Reverse: GATCAGTTGTCTCTGGTTGCAAATC). Human β-globin (Forward: CAACTTCCACGGTTCACC; Reverse: GAAGAGCCAAGGACAGGTAC) was used as an internal control. For the determination of HPV-16 E2 and E6/7 viral loads, HPV-16 DNA standard calibration curves were generated by plotting the threshold cycle (Ct values) against the logarithm of the copy number of seven-fold serially diluted Caski DNA with spiked HPV-16 negative HNC cell line DNA (SCC-25). The qPCR used the following conditions: 50 °C for 10 min; 95 °C for 10 min; 40 cycles at 95 °C for 15 s and 60 °C for 60 s; and a final melting curve analysis with following conditions: 95 °C for 15 s, 60 °C for 60 s, and 95 °C for 15 s.

### 4.5. HPV-16 Physical Status

HPV-16 physical status in saliva samples collected from HPV-16 DNA positive OPC patients (*n* = 71) was determined as described previously [37]. HPV-16 was classified as an episomal form when the E2/E6/7 ratio was equal to or more than 1, a fully integrated form when the E2/E6/7 ratio was equal to 0, and a mixed form when the E2/E6/7 ratio was between 0 and 1.

### 4.6. Statistical Analysis

The inter-rater agreement between tumor p16 expression and oral HPV-16 infection was determined by the Cohen’s kappa coefficient with a 95% confidence interval (https://www.graphpad.com/quickcalcs/kappa1.cfm). Sensitivity, specificity, positive predictive values (PPV), negative predictive values (NPV), and their 95% confidence intervals were calculated. The potential association between HPV-16 DNA viral load/physical status and the disease stage of OPC patients was examined by the Fisher’s exact test, and p values less than 0.05 were considered significant. All of the statistical analysis were performed using GraphPad Prism 7 software version 7 (GraphPad Software Inc., San Diego, CA, USA).

## 5. Conclusions

Taken together, our data supports the use of salivary HPV DNA as a non-invasive biomarker in OPC patients, and indicates that disease staging could be based on viral load in combination with an eighth edition staging system.

## Figures and Tables

**Table 1:** Patient demographics and characteristics.

Variables	Categories	OPC (*N* = 121)	
		No.	%
Age (Years)	≤55	31	25.6
	>55	90	74.4
Sex	Male	110	90.9
	Female	11	9.1
Smoking Status	Ever	89	73.6
	Never	31	25.6
	Unknown	1	0.8
Drinking Status	Ever	59	48.8
	Never	61	50.4
	Unknown	1	0.8
Anatomical Site	Tonsil	60	49.6
	Base of Tongue	37	30.6
	Both	8	6.6
	Others	16	13.2
p161INK4a Status	Positive	89	73.6
	Negative	32	26.4
Salivary HPV-16 DNA Status	Positive	74	61.2
	Negative	47	38.8

**Table 2:** Tumor p16 expression and oral HPV-16 infection in OPC. OPC patients (*n* = 121).

Salivary HPV-16 DNA Status	p16 Status	
	Positive	Negative
Positive	71 (80%)	3 (9%)
Negative	18 (20%)	29 (91%)

Sensitivity 0.80 (0.70, 0.87), Specificity 0.91 (0.76, 0.97), Positive Predictive Value (PPV) 0.96 (0.89, 0.99), Negative Predictive Value (NPV) 0.62 (0.47, 0.74).

**Table 3:** Salivary HPV-16 physical status in OPC patients. HPV-16 DNA-positive OPC patients (*n* = 71).

HPV-16 Physical Status	7th Edition				8th Edition			
	State I/II		State III/IV		State I/II		State III/IV	
	No.	%	No.	%	No.	%	No.	%
Episomal	1	20.0	22	33.3	17	30.4	6	40.0
Mixed/Integrated	4	80.0	44	66.7	39	69.6	9	60.0
	*p* = 1.0				*p* = 0.541			
